# Nose-to-brain siRNA delivery by PEI/PPI-based nanoparticles reduces α-synuclein expression in a Parkinson’s disease mouse model

**DOI:** 10.1016/j.omtn.2025.102671

**Published:** 2025-08-06

**Authors:** Malte Feja, Isabell Drath, Sandra Weiß, Alexander Ewe, Birthe Gericke, Tiago F. Outeiro, Leonidas Stefanis, Achim Aigner, Franziska Richter

**Affiliations:** 1 Department of Pharmacology, Toxicology, and Pharmacy, University of Veterinary Medicine Hannover, Hanover, Germany; 2 Center for Systems Neuroscience (ZSN), Hannover, Germany; 3 Rudolf-Boehm-Institute for Pharmacology and Toxicology, Clinical Pharmacology, Faculty of Medicine, University of Leipzig, Leipzig, Germany; 4 University Medical Center Göttingen, Department of Experimental Neurodegeneration, Center for Biostructural Imaging of Neurodegeneration, Göttingen, Germany; 5 First Department of Neurology, Eginition Hospital, National and Kapodistrian University of Athens Medical School, Athens, Greece

**Keywords:** MT: Delivery Strategies, intranasal, *in vivo* siRNA delivery, polyethylenimine, polypropylenimine, RNA interference, Thy1-aSyn mice

## Abstract

Potential strategies to develop new treatments for Parkinson’s disease (PD) aim at targeting disease-associated proteins like alpha-synuclein (aSyn), which accumulates in neurons of PD patients and contributes to neuronal degeneration. A promising new approach is the therapeutic use of small interfering RNAs (siRNAs) for aSyn knockdown, but is challenging due to siRNA instability, poor delivery, and inefficient uptake. Therefore, we developed a nanoparticle-based approach for intranasal delivery of siRNAs, circumventing the blood-brain barrier and enhancing the potential of siRNAs for clinical application. Tyrosine-modified polyethylenimines (PEIs), or polypropylenimine dendrimers (PPIs), were complexed with siRNA targeting the aSyn-encoding gene *SNCA* (siSNCA) and combined with liposomes. Nanoparticles efficiently transfected SH-SY5Y cells with low cytotoxicity and significantly reduced *SNCA* mRNA levels. In Thy1-aSyn mice, intranasally administered labeled nanoparticles distributed extensively across the brain, including the olfactory bulb, substantia nigra, and prefrontal cortex. After only 4 days of treatment, siSNCA-loaded nanoparticles significantly reduced aSyn protein and *SNCA* mRNA levels in the brain. Mice showed neither overt adverse behavioral effects nor increased reactive microglia. These findings highlight the potential of nanoparticle-mediated intranasal siRNA delivery as a promising, non-invasive approach to reduce aSyn levels in the brain, offering a novel therapeutic strategy for Parkinson’s disease.

## Graphical abstract

**Figure:**
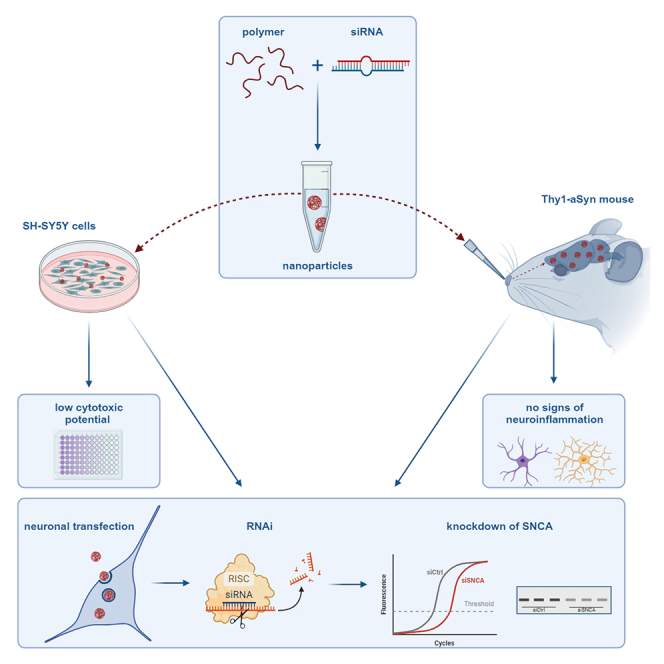

## Teaser

Intranasal nanoparticle delivery of siRNA effectively reduces brain alpha-synuclein levels in a Parkinson’s disease mouse model, offering a non-invasive therapeutic strategy with clinical potential.

## Introduction

Parkinson`s disease (PD) is a progressive, incurable disorder characterized by the loss of dopaminergic neurons in the substantia nigra pars compacta (SNc) that leads to motor impairments.^1^ Current therapy is limited to symptomatic treatment of motor dysfunction, but fails to prevent or slow neurodegeneration and does not address non-motor symptoms.^2^^,^^3^ These limitations highlight the need for novel therapeutic approaches. Potential strategies to develop novel treatments aim at targeting specific proteins and related pathways with demonstrated relevance to PD, e.g., alpha-synuclein (aSyn) that accumulates and aggregates in neurons of PD patients, contributing to cell degeneration.^4^ The occurrence of aSyn accumulations in the midbrain, including the substantia nigra, correlates with severity of Parkinson’s symptoms, indicating a connection to the neuronal dysfunction of patients.^5^ Moreover, aSyn has been shown to disrupt mitochondrial function in neurons^6^ and activate microglia leading to an inflammatory response in various brain regions.^7^^,^^8^ Accordingly, one of the most promising therapeutic approaches to date is the reduction of aSyn accumulation and its downstream effects.

A potential method for lowering aSyn protein levels involves the use of small interfering RNA (siRNA) targeting the messenger (m)RNA of the aSyn-encoding gene (*SNCA*). RNA interference (RNAi) has promising therapeutic value due to its ability to silence specific genes and modulate protein expression. However, *in vivo* delivery of small RNAs to neurons remains challenging due to RNA instability, fast degradation,^9^ the need to bypass the blood-brain barrier, and their low transfection efficacy.^10^ Since 2018, five siRNA drugs have received approval for clinical use by the US Food and Drug Administration and the European Medicines Agency, all primarily targeting the liver.^11^ However, efficient delivery to non-liver tissues, particularly the brain, which is required for the treatment of CNS disorders such as PD, continues to present considerable challenges in siRNA drug development. Consequently, there is need for appropriate delivery formulations and routes. In the field of neurodegenerative diseases, adeno-associated viruses are commonly used for gene delivery, but they have disadvantages such as limited loading capacity, difficulties in vector production, and potential inflammatory reactions.^12^^,^^13^ Therefore, non-viral carriers including liposomal and polymeric nanoparticles are considered as alternatives for nucleic acid delivery.^14^ We previously provided initial proof of principle for the efficacy of nanoparticle-mediated delivery of siRNA targeting *SNCA* mRNA (siSNCA) to the brain for therapeutic intervention. Intracerebroventricular injections of siSNCA complexed into polymeric, polyethylenimine (PEI)-based nanoparticles reduce brain *SNCA* mRNA levels by up to 65%^15^ in a mouse model overexpressing human wild-type aSyn under the murine Thy-1 promoter (Thy1-aSyn mice).^16^^,^^17^ Besides the low-molecular weight, branched PEI F25, we previously established further advanced nanocarriers, such as linear or branched low-molecular weight, tyrosine-modified PEIs as well as tyrosine-modified polypropylenimine (PPI) dendrimers for highly efficient siRNA delivery *in vitro* and *in vivo*.^18^^,^^19^^,^^20^^,^^21^^,^^22^^,^^23^^,^^24^^,^^25^^,^^26^ The tyrosine-modification results in higher complex stability, improved cellular uptake, transfection efficacy, and higher biocompatibility, but success strongly depends on polymer properties, e.g., molecular weight and branched or linear structures.^20^^,^^21^^,^^24^^,^^27^ Additionally, we previously established combinations of PEI F25-based polyplexes with liposomes, leading to lipopolyplexes.^25^ We could show that these lipopolyplexes (LPPs) combine physicochemical properties of both components, leading to high efficacy and lower aggregation tendency.^28^ Given the promising outcomes we have achieved *in vitro* and *in vivo* within tumor models with the modified PEI/PPI-based polymers on the one hand and the combination of polyplexes with liposomes on the other hand,^19^^,^^21^^,^^25^ our focus now is to explore whether and to what extent the modified nanoparticles are suitable for transporting siRNA to the brain in models of neurodegeneration.

Direct application methods into the brain, such as convection-enhanced drug delivery via intracranial microinjections or implantable drug pumps, effectively bypass the BBB, but are invasive.^29^ Nose-to-brain delivery provides a direct route to the brain, circumventing the blood-brain barrier and rendering it suitable for daily use.^30^^,^^31^ Pharmacokinetic profiles have revealed superior efficiency in brain delivery of siRNA combined with nano-sized micelles via intranasal application compared with intravenous administration in rats.^32^ Therefore, we aimed to develop an intranasal nanoparticle-mediated gene therapy strategy for the treatment of PD to deliver siRNA targeting human *SNCA* to the brain and thereby intervene in the progression of aSyn pathology in the Thy1-aSyn mouse model. Our study explored a panel of PEI/PPI-based polymeric nanoparticle systems, and LPP derivatives thereof. Polymers include unmodified PEI F25, low-molecular weight, tyrosine (Y)-modified branched and linear PEIs (P10Y and LP10Y), as well as the tyrosine-modified polypropylenimine (PPI) dendrimer PPI-Y. Polyplexes (PPs) and LPPs were loaded with siRNA for neuronal uptake, biocompatibility, and aSyn knockdown efficacy in SH-SY5Y cells and after intranasal application in Thy1-aSyn mice.

## Results

### PEI/PPI-based nanoparticles reveal low cytotoxic potential and polymer-dependently knock down *SNCA* mRNA *in vitro*

**Figure 1:**
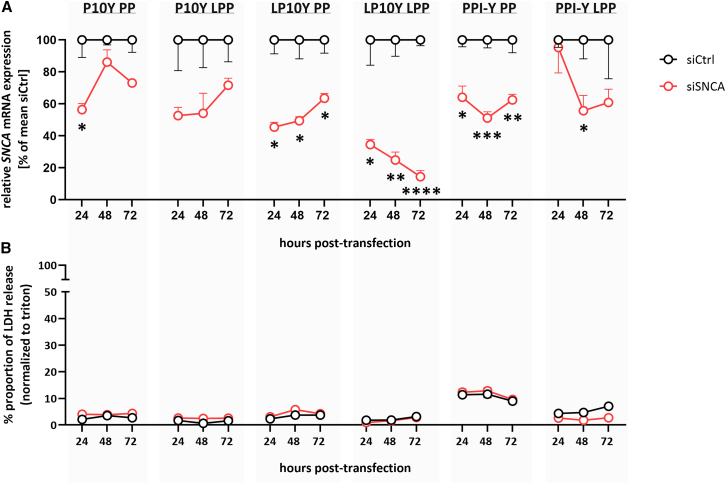
Knockdown of *SNCA* mRNA and cytotoxicity by polyethylenimine (PEI)/polypropylenimine (PPI)-based nanoparticles in SH-SY5Y alpha-synuclein (aSyn) overexpressing cells Negative control small interfering RNA (siCtrl) or siRNA targeting the mRNA of aSyn (siSNCA) were complexed into tyrosine-modified branched (P10Y) and linear PEI (LP10Y) or tyrosine-modified PPI (PPI-Y) polyplexes (PP) or lipopolyplexes (LPP). The effects of siSNCA/siCtrl-loaded nanoparticles 24, 48, and 72 h after transfection (*n* = 3 wells per condition) on (A) *SNCA* mRNA expression and (B) the proportion of lactate dehydrogenase (LDH) release are shown as mean +/− SEM. 75 pmol of siRNA/well on a six-well plate and 15 pmol siRNA/well on a 96-well plate were used for the knockdown experiment and cytotoxicity (LDH) assay, respectively. Results are presented as relative values: in (A), calculated as percent of mean siCtrl, using *actb* as reference gene, and in (B), normalized to Triton X-100 (100% cell death, not shown). Values represent individual experiments performed in technical triplicates (biological replicates). Two-way ANOVA with Geisser-Greenhouse correction and Sidak’s multiple comparisons for post hoc analysis; ∗*p* < 0.05, ∗∗*p* < 0.01, ∗∗∗*p* < 0.001, ∗∗∗∗*p* < 0.0001.

Besides PEI F25 PP and LPP previously validated for siRNA delivery *in vitro* and *in vivo*,^15^^,^^33^^,^^34^ six other nanoparticles based on chemically modified polymers were employed. Differences in hydrodynamic diameters, as determined by dynamic light scattering (DLS), were seen between the different polymers and between the different buffers used for complexation, i.e., HN buffer vs. trehalose/glucose buffer (Figures S1A and S1B; bars). Likewise, electrophoretic light scattering (ELS) revealed major differences in zeta potentials of the complexes (Figures S1A and S1B; dots). Agarose gel electrophoresis of complexed and free siRNA also revealed full complexation of the siRNA at the selected polymer/siRNA ratios, as seen from the absence of the free siRNA band in the nanoparticle samples (Figure S1C). Based on the observation of larger nanoparticle sizes after complexation in HN buffer and the presence of smaller nanoparticles when complexed in trehalose buffer, the latter were chosen for subsequent *in vivo* experiments, while complexation in HN buffer was employed for *in vitro* studies. Uptake experiments in comparably hard-to-transfect differentiated SH-SY5Y neuroblastoma cells showed efficient cell internalization of all nanoparticles (exemplified in Figure S2). This was seen after 4-h and 24-h incubation (data not shown) and provided the basis for further evaluating their potential for targeting *SNCA* mRNA *in vitro*. All tyrosine-modified PEI/PPI complexes containing siSNCA achieved at least a 40% reduction in *SNCA* mRNA expression compared with negative siCtrl (siRNA against luciferase) within 72-h incubation in SH-SY5Y Tet-Off aSyn-overexpressing cells (Figure 1A). Complexes based on LP10Y and PPI-Y were most efficient, with LP10Y as lipopolyplex showing the most pronounced knockdown efficacy across all time points, peaking at an almost complete reduction in *SNCA* mRNA levels (F_(1, 4)_ = 2807, *p* < 0.0001; 86%) 72 h after transfection. As a polyplex, LP10Y and PPI-Y induced a robust *SNCA* mRNA knockdown as well that was maintained over the entire 72-h incubation period (LP10Y PP: F_(1, 4)_ = 187.9, *p* = 0.0002; up to 55%; PPI-Y PP: F_(1, 4)_ = 210.3, *p* = 0.0001; up to 49%). Transfection with PPI-Y lipopolyplexes and P10Y-based nanoparticles containing siSNCA resulted in a comparable ∼45% knockdown of *SNCA* mRNA (PPI-Y LPP: F_(1, 4)_ = 30.83, *p* = 0.0051; P10Y PP: F_(1, 4)_ = 123.1, *p* = 0.0004).

In addition to efficient knockdown activity, high biocompatibility is a crucial prerequisite for nanoparticles to be considered as potential candidates for therapeutic use.^21^ Therefore, we next determined the potential cytotoxicity of the selected nanoparticles in SH-SY5Y Tet-Off aSyn-overexpressing cells. Lactate dehydrogenase (LDH) release assays showed very low values for the nanoparticles (Figure 1B). The vast majority of the complexes produced an LDH release of ∼5% within 72 h of incubation, indicating the absence of acute cytotoxicity.^35^ PPI-Y polyplexes showed slightly elevated LDH release levels around 10%, which is still considered non-toxic in this assay.^35^ Taken together, all PEI/PPI-based nanoparticles demonstrated excellent biocompatibility, independent of the loaded siRNA.

### PEI/PPI-based nanoparticles applied to the nose are distributed in the brain and taken up by neurons

**Figure 2:**
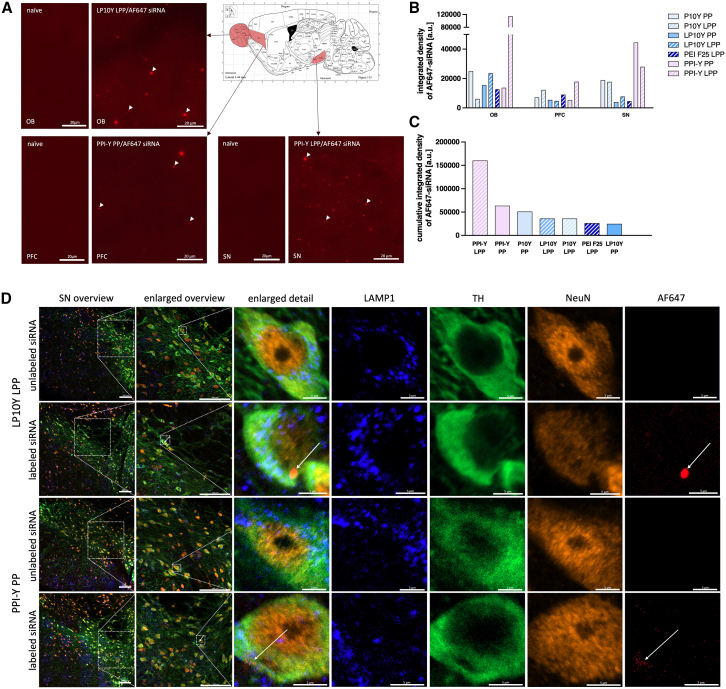
Distribution and neuronal uptake of polyethylenimine (PEI)/polypropylenimine (PPI)-based nanoparticles in the central nervous system Alexa Fluor (AF) 647-labeled small interfering (si)RNA was complexed with unmodified branched PEI F25, with tyrosine-modified branched (P10Y) and linear PEIs (LP10Y) or with tyrosine-modified PPI (PPI-Y) into polyplexes (PP) or lipopolyplexes (LPP). (A) Microscopy of coronal brain sections revealed red fluorescence of AF647-siRNA in the olfactory bulb (OB), prefrontal cortex (PFC), and substantia nigra (SN) after intranasal administration (4 days, once daily, 300 pmol siRNA, *n* = 1 per nanocarrier) in 2-month-old Thy1-aSyn mice. White arrows indicate location of AF647-siRNA. 400× total magnification, scale bar, 20 μm. (B) Integrated density of AF647-siRNA signal in OB, PFC, and SN after nanoparticle application. Integrated density background values for a naive mouse brain are 65.12 in OB, 43.84 in PFC and 61.74 in SN. (C) Cumulative AF647-siRNA signal in OB, PFC, and SN. Cumulative integrated density of background signal in a naive mouse brain is 170.69. (D) Confocal microscopy of coronal brain sections visualized fluorescence of AF647-conjugated control small interfering RNA (siCtrl, 300 pmol) loaded into PPI-Y PP and LP10Y LPP in tyrosine hydroxylase (TH)-positive neurons of the SN, indicating uptake into dopaminergic neurons after intranasal administration (4 days, once daily; see also Figure S3) in 6-month-old mice. Negative control, nigral sections of mice treated with unlabeled siRNA-LP10Y/PPI-Y complexes showed no fluorescent signal in the far-red spectrum. Blue, lysosomal-associated membrane protein 1 (LAMP1); green, TH; orange, NeuN as neuronal marker; 250× total magnification; scale bar, 5 μm (overview image: 100 μm). White arrows indicate location of AF647-siRNA.

To analyze gene silencing efficiency of the different nanoparticles, we used male Thy1-aSyn mice, which overexpress the siRNA target gene, human wild-type *SNCA*, in neurons. Before promising delivery platforms like PEIs or PPIs can be used for therapeutic nucleic acid administration, *in vivo* assessment of key characteristics that are crucial for clinical applicability, such as tissue penetration and biodistribution, is necessitated.^24^ To determine the successful intranasal administration of PEI- or PPI-based nanoparticles for siRNA delivery and their distribution in the central nervous system (CNS) of Thy1-aSyn mice, coronal brain sections were both qualitatively and quantitatively analyzed for fluorescent signal presence following 4 days of AF647-siRNA loaded nanoparticle administration (20 μL/nostril once daily; *n* = 1 per nanoparticle variant). We found that all tested nanoparticles (including PEI F25) reached the brain after intranasal application. They distributed extensively across the brain and were detectable in application-near, i.e., olfactory bulb, prefrontal cortex, as well as more distal regions, such as substantia nigra (Figure 2A). It is important to note that only one animal per nanoparticle formulation was analyzed in this experiment. Accordingly, the observed distribution patterns are intended to be qualitative and representative, not quantitative or comparative. Signal intensity and regional localization varied between formulations: some nanoparticles exhibited stronger fluorescence in the olfactory bulb (OB), suggesting local accumulation near the application site, while others showed detectable signals in deeper regions such as the substantia nigra (SN), indicating broad tissue penetration (Figures 2B and 2C). This feasibility-focused approach, using one animal per formulation, aligns with a prior foundational study,^15^ in which CNS-wide biodistribution of PEI/siRNA complexes after intracerebroventricular injection was likewise demonstrated in a single animal.

**Figure 3:**
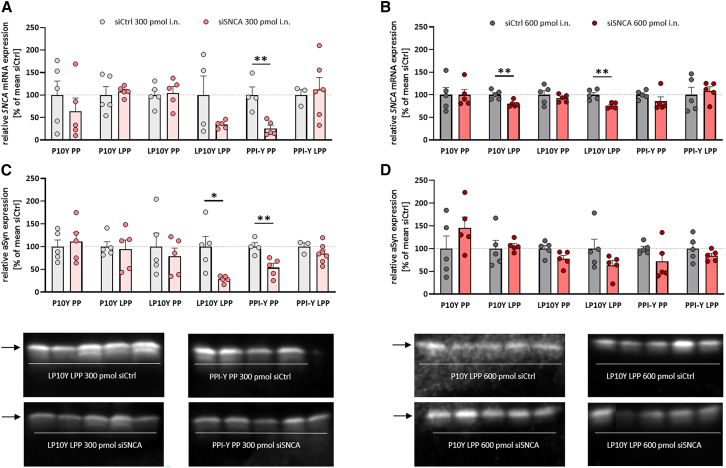
Knockdown of *SNCA* mRNA and alpha-synuclein (aSyn) by siRNA targeting the mRNA of aSyn (siSNCA) complexed into polyethylenimine (PEI)/polypropylenimine (PPI)-based nanoparticles Control small interfering RNA (siCtrl) or siSNCA were loaded into tyrosine-modified branched (P10Y) and linear PEI (LP10Y) or tyrosine-modified PPI (PPI-Y) polyplexes (PP) or lipopolyplexes (LPP). The effects of siSNCA/siCtrl-loaded nanoparticles after intranasal application (4 days, once daily, 300 pmol and 600 pmol siRNA, n = 3–7 mice/group, 2-month-old mice) on the relative *SNCA* mRNA expression in the posterior half of the brain (A and B; using actb as reference gene) and aSyn expression in relation to total protein in the anterior half of the brain (C and D; see also Figures S4 and S5) are shown as mean + SEM. The results are presented as relative values calculated as percent of mean siCtrl. Immunoblots with Syn1 as primary antibody and bands corresponding to aSyn (arrows at 17 kDa) are shown for respective treatment groups. Unpaired t tests with Welch’s correction in case of unequal variances; ∗*p* < 0.05, ∗∗*p* < 0.01.

To confirm cellular uptake of the nanoparticles after brain penetration, the polymers that demonstrated the highest *SNCA* mRNA knockdown efficacy *in vitro* (i.e., LP10Y as a lipopolyplex and PPI-Y as a polyplex) were used for complexation of AF647-labeled siRNA (siCtrl, 300 pmol) or unlabeled siSNCA (here acting as negative control; 300 pmol) and administered intranasally to mice on 4 consecutive days. Via confocal microscopy of coronal brain sections, fluorescent signal was observed in tyrosine hydroxylase (TH) and NeuN-positive cells in the SNc, primarily in the perinuclear zone, implying the uptake of siRNA into dopaminergic neurons (see Figure 3D). Signals from AF647 were also found to colocalize with the lysosomal-associated membrane protein (LAMP)1, suggesting the subcellular localization of siRNA in the lysosomes. TH-positive cells also demonstrated AF647 red fluorescence that was not colocalized with LAMP1 and NeuN, indicating the presence of released siRNA in the cytoplasm. In addition, we detected colocalization of AF647 with NeuN and beta-III-tubulin, another neuronal marker, indicating uptake of AF647-siRNA into neuronal nuclei (see Figure S3). As a negative control, nigral sections from mice administered unlabeled siRNA-LP10Y/PPI-Y complexes showed no fluorescence signal in the far-red spectrum. In summary, LP10Y LPP and PPI-Y PP show successful delivery of siRNA into the brain with subsequent neuronal uptake after intranasal application in Thy1-aSyn mice.

### PEI/PPI-based nanoparticles show polymer-dependent *SNCA* knockdown efficacy *in vivo* at the mRNA and protein level

To determine the biological efficacy of the nanoparticles based on the more recently developed advanced polymers, the different (lipo)polyplexes were loaded with siRNA against *SNCA* or unrelated siRNA against luciferase (negative control) at doses of 300 pmol and 600 pmol (corresponding to an amount of 4 μg and 8 μg siRNA, respectively) and intranasally applied (once daily for 4 days) to Thy1-aSyn mice. *SNCA* mRNA levels in the dissected posterior half of the brain were determined by qRT-PCR. Nose-to-brain delivery of siSNCA in Thy1-aSyn mice resulted in a polymer- and dose-dependent knockdown of *SNCA* mRNA (Figures 3A and 3B). LP10Y LPP and PPI-Y PP showed profound efficacy at 300 pmol siSNCA, causing a 66% and a significant 74% knockdown of *SNCA* mRNA (*p* = 0.0041), respectively, compared with siCtrl (Figure 3A). The P10Y/siSNCA polyplex showed a moderate, albeit non-significant, knockdown efficacy of 36% on *SNCA* mRNA. In contrast, P10Y LPP, LP10Y PP, and PPI-Y LPP showed no knockdown effects on mRNA expression compared with the negative control. At 600 pmol siSNCA, LP10Y LPP significantly reduced *SNCA* mRNA levels by 24% (*p* = 0.003), whereas the 15% reduction induced by PPI-Y PP was not significantly different from the control group (Figure 3B). While P10Y LPP was not efficient at low siSNCA dose, it significantly decreased *SNCA* mRNA levels by 20% at the higher siSNCA dose. P10Y PP, LP10Y PP, and PPI-Y LPP had no effect on *SNCA* mRNA expression at 600 pmol siSNCA.

Knockdown of *SNCA* mRNA upon intranasal treatment of mice with 300 pmol siSNCA loaded into LP10Y LPP and PPI-Y PP also resulted in less *SNCA* protein, as determined by western blot analysis of aSyn amounts in the anterior half of the brain (Figures 3C and 3D). Specifically, LP10Y-lipopolyplexed and PPI-Y polyplexed siSNCA significantly decreased the amount of aSyn relative to the total protein by 72% (*p* = 0.0323) and 46% (*p* = 0.0089), respectively, compared with control siRNA (Figure 3C), indicating successful silencing of *SNCA* overexpression. LP10Y polyplexed and PPI-Y lipopolyplexed siSNCA (300 pmol) resulted in a 21% and 15% reduction in aSyn expression, respectively, which was not significantly different from control siRNA. The mRNA knockdown induced by the higher dose of siSNCA (600 pmol) was also observed at the protein level for LP10Y LPP (37% aSyn reduction) and PPI-Y PP (38% aSyn decrease), but not in the case of P10Y LPP (Figure 3D; see also Figure S5). LP10Y polyplexed and PPI-Y lipopolyplexed siSNCA reduced relative aSyn expression by 23% and 16%, respectively, although the effects did not reach significance level. P10Y polyplexed siSNCA at 600 pmol showed no knockdown efficacy at the protein level. Since the Syn1 primary antibody detects both murine and human aSyn, we tested the knockdown efficacy with another primary mouse monoclonal antibody (Syn211) that is specific for human aSyn.^36^ A profound reduction of aSyn expression by LP10Y LPP (60%, *p* = 0.0357 at 300 pmol; 34%, *p* = 0.0551 at 600 pmol) and PPI-Y PP (46% at 300 pmol; 24% at 600 pmol) at both siSNCA doses was again seen. The analysis also unveiled knockdown efficiency for P10Y-based polyplexes (74%, *p* = 0.0207) and lipopolyplexes (48%, *p* = 0.0886) at 300 pmol and for PPI-Y LPP (44%) at 600 pmol siSNCA dose (see Figures S4 and S5). These findings substantiate the knockdown efficacy, especially of LP10Y LPP and PPI-Y PP, against human aSyn.

### Nose-to-brain delivery of PEI/PPI-based nanoparticles does not trigger neuroinflammation

**Figure 4:**
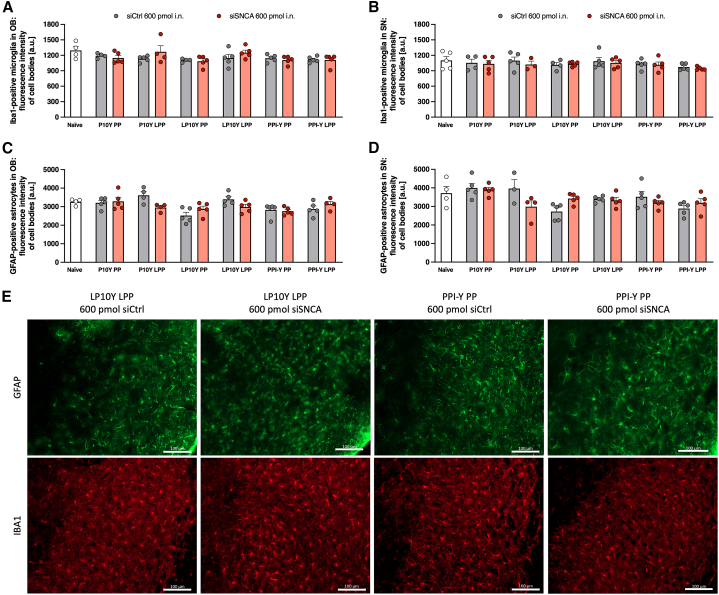
Nose-to-brain delivery of polyethylenimine (PEI)/polypropylenimine (PPI)-based nanoparticles does not induce neuroinflammation Control small interfering RNA (siCtrl) or siRNA targeting the mRNA of alpha-synuclein (siSNCA) were loaded into tyrosine-modified branched (P10Y) and linear PEI (LP10Y) or tyrosine-modified PPI (PPI-Y) polyplexes (PP) or lipopolyplexes (LPP). The effects of intranasal application of siSNCA/siCtrl-loaded nanoparticles (once daily for 4 days, 600 pmol siRNA, n = 3–5 mice/group) on the fluorescence intensity of Iba1-positive microglial cell bodies in (A) the olfactory bulb (OB) and (B) substantia nigra (SN) and of GFAP-positive astrocytic cell bodies in (C) OB and (D) SN of 2-month-old Thy1-aSyn mice are shown as mean + SEM. Untreated Thy1-aSyn mice served as naive control. Two-way ANOVA with Geisser-Greenhouse correction and Sidak’s multiple comparisons for post hoc analysis; n.s., *p* < 0.05 naive vs. siCtrl/siSNCA, siCtrl vs. siSNCA. (E) Immunofluorescence staining of Iba1-positive microglia (red) and GFAP as a marker for astrocytes (green) in the substantia nigra of Thy1-aSyn mice treated with LP10Y LPP and PPI-Y PP loaded with siCtrl or siSNCA. 200× total magnification, scale bar, 100 μm.

To determine whether nasal administration of PEI/PPI-based nanoparticles into the brain induces intracerebral neuroinflammatory responses, we quantitatively assessed Iba1-positive microglia and glial fibrillary acidic protein (GFAP)-positive astrocytes as well as mRNA levels of the cytokines interleukin (IL)-1β, IL-6, and tumor necrosis factor (TNF)-α after intranasal administration of the higher siRNA dose (600 pmol) in Thy1-aSyn mice. We observed no increase in reactive microglia (Figures 4A, 4B, and 4E) or astrogliosis (Figures 4C–4E) in the OB and SN in PEI/PPI-siSNCA-treated mice compared with negative (siCtrl) or naive, untreated controls. Moreover, for all three measured cytokines (IL-1β, IL-6, and TNF-α) the Ct values were at 35 or above (Figure S7), supporting absence of cytokine response in the brain across all groups. The Ct of the housekeeping gene beta actin was on average 25, thus the cytokine threshold was 10 times above, further supporting absence of significant cytokine expression. Thus, *SNCA* knockdown induced by PEI/PPI-based nanoparticles was not found to be associated with neuroinflammation. Furthermore, nanoparticles loaded with 300–600 pmol siRNA were well tolerated by Thy1-aSyn mice and had no effect on general health or body weight.

## Discussion

The primary objective of this study was to develop an innovative, non-invasive delivery strategy for a gene therapeutic approach to silence aSyn expression in the brain as a treatment option for PD. We provide proof-of-concept that brain delivery of siRNA targeting human *SNCA* to intervene in the progression of aSyn pathology can be achieved by intranasal application of PEI- or PPI-based, chemically modified nanoparticles in the Thy1-aSyn mouse model of PD. This study presents four key findings: first, nanoparticles composed of PEI/PPI-siRNA complexes reveal excellent biocompatibility and effectively knock down *SNCA* mRNA expression in aSyn overexpressing SH-SY5Y neuroblastoma cells. Second, intranasally applied PEI/PPI-based nanoparticles are widely distributed in the brain and efficiently taken up by dopaminergic SNc neurons in Thy1-aSyn mice. Third, nose-to-brain delivery of siRNA by PEI/PPI-based nanocarriers reduces SNCA mRNA and protein levels in our PD mouse model, and fourth, does not trigger neuroinflammation.

Despite the unclear initial triggers of pathology and progression in neurodegenerative synucleinopathies, such as PD, characterized by deposited aggregates of misfolded neuronal aSyn in the brain,^37^ there is ample evidence supporting therapeutic targeting of aSyn to limit its prion-like misfolding.^38^ With the increasing attention toward nucleic acid-based therapeutics for PD, RNAi emerges as a promising gene therapeutic approach in reducing aSyn and its progressive pathology.^39^ However, *in vivo* RNAi applications face two major challenges: to protect siRNAs from degradation and to transport them to the site of action in the target tissue cells.^11^^,^^40^ Considering that the target cell supplies all other components of the RNAi machinery, the efficiency of siRNA delivery has proven to be the limiting factor for successful RNAi application.^41^

In the present study, all different PEI-based nanoparticles (branched P10Y and linear LP10Y, both as polyplexes and lipopolyplexes) proved to be internalized by differentiated SH-SY5Y neuroblastoma cells. Among non-viral systems, highly branched, cationic PPIs have emerged as promising siRNA carriers.^42^ Here, PPI-based nanocarriers (PPI-Y dendriplex and lipodendriplex) also mediated successful *in vitro* cellular internalization of siRNA. This was seen despite their rather large sizes with diameters of almost 600 nm at mass ratio 2.5 and their very low surface charge, thus being in line with previous studies on tyrosine-modified PPI dendrimers that had shown efficient siRNA cellular internalization in human lung and prostate cancer cell lines.^24^

Improved cell uptake of the tyrosine-modified PEI/PPI-based nanoparticles was shown previously to lead to enhanced biological efficacy *in vitro*.^24^ Indeed, in the present study, all tyrosine-modified PEI/PPI-based complexes containing siSNCA showed high transfection efficacy in SH-SY5Y cells, leading to the profound reduction in *SNCA* mRNA expression within 72 h of *in vitro* incubation.

Beyond biological activity, nanoparticle biocompatibility is pivotal for therapeutic use. This is particularly true for positively charged nanoparticles that are generally associated with higher cytotoxicity. We found that all PEI/PPI-based nanoparticles displayed very high biocompatibility over a prolonged 72-h incubation period in SH-SY5Y aSyn-overexpressing cells. Previously, polyplexes based on P10Y, LP10Y, and PPI-Y proved to be highly biocompatible *in vitro* by showing less than 10% release in LDH assays in ovarian^24^ and prostate carcinoma cells.^20^ PPI-Y dendriplexes showed better biocompatibility in primary mesenchymal stromal cells^43^ and prostate carcinoma cells^24^ compared with P10Y and LP10Y polyplexes.

While the strongly positive charge of cationic polymer-based complexes allows efficient nucleic acid complexation and cellular uptake, it also contributes to colloidal instability under physiological conditions, promoting complex aggregation.^44^ To improve the *in vivo* delivery of therapeutic nucleic acids, these complexes can be combined with liposomes, forming lipopolyplexes that exhibit increased *in vitro* transfection efficiency as well as an improved cytotoxic profile.^45^^,^^46^ Lipopolyplexes consisting of PEI F25 and the phospholipid DPPC had shown high siRNA transfection efficiency for siRNA and reduced surface charges in SKOV-3 cells^47^ and excellent biocompatibility in Tu2449 cells,^23^ suggesting favorable *in vivo* application properties. This was now extended toward the chemically modified polymers. Consistent with these findings, PPI-Y LPP demonstrated a further improvement of the already low cytotoxic profile observed for the PPI-Y polyplex. It has been noted in past studies that linear PEIs exhibit higher toxicity compared with their branched counterparts.^22^^,^^48^ Here, both branched and linear PEIs displayed minimal observable cytotoxicity, likely due to the positive effect of tyrosine grafting. From the *in vitro* results obtained, we can conclude that the nanoparticles studied here exhibited very low cytotoxicity. The successful internalization of the PEI/PPI-based nanocarriers into SH-SY5Y cells indicates their potential for safely delivering siRNA into the brain. This suggests their potential efficacy in treating CNS disorders such as PD.

Since efficient uptake in a cultured cell does not necessarily equate to an efficient uptake *in vivo*, we evaluated critical attributes for clinical application, such as tissue penetration and biodistribution, in a mouse model that reproduces key features of sporadic PD. After intranasal administration of PEI/PPI-siRNA complexes in Thy1-aSyn mice, all examined nanoparticle types were found to reach the brain, where they were widely distributed, including regions such as the olfactory bulb, prefrontal cortex, and substantia nigra. We have previously shown that PEI F25-siRNA complexes distribute widely across the CNS after direct intracerebroventricular injection in Thy1-aSyn mice.^15^ Notably, in the present study the other nanoparticles mostly displayed enhanced brain distribution compared with PEI F25, suggesting improved tissue penetration capabilities of these formulations. Specifically, nanoparticles based on PPI-Y exhibited a wide brain distribution, with the PPI-Y lipopolyplex presenting the highest prevalence, indicative of its substantial tissue penetration capacity. Although most *in vivo* studies tend to utilize smaller nanoparticles considered more appropriate for tissue penetration upon systemic administration, our findings are supported by a previous study demonstrating the benefit of larger complexes for intratumoral injections in subcutaneous xenografts or abdominal tumor models.^21^ While definitive conclusions about distribution efficiency cannot be drawn from these single-animal observations, we can qualitatively determine which brain regions have been reached by the siRNA and provide an initial assessment of which nanoparticles appear particularly effective at delivering siRNA to the brain. Since all tested nanoformulations were capable of reaching multiple brain regions after intranasal administration, our findings support the overall feasibility of this non-invasive delivery route for siRNA-based therapeutics targeting the CNS. As the main readout parameter is knockdown efficiency, we decided to keep the number of animals used for biodistribution low in alignment with the principles of the 3Rs. However, a more comprehensive quantitative assessment of biodistribution would require a higher number of animals and a bulk analysis method. Our findings suggest that PEI/PPI-based nanoparticles enable substantial siRNA distribution in the mouse brain. In particular, the nanoparticles are sufficiently stable to fully protect the siRNA against premature release and thus degradation and to mediate cellular internalization, while at the same time allowing intracellular siRNA release for subsequent RNA-induced silencing complex (RISC) incorporation and knock down activity. This highlights their potential as a non-invasive delivery platform for therapeutic applications targeting the CNS. This is particularly relevant for PD, where *SNCA* pathology is not limited to a single brain region but spreads throughout the CNS as the disease progresses. Notably, the cardinal motor symptoms of PD arise from a specific loss of dopaminergic neurons in the substantia nigra.^5^ Using LP10Y LPP and PPI-Y PP, we established that PEI/PPI-based nanoparticles can successfully deliver siRNA into nigral dopaminergic neurons in Thy1-aSyn mice. The siRNA was predominantly localized to the perinuclear cytoplasm, consistent with the primary site of RNA interference activity mediated by the cytoplasmatic RISC. A weaker siRNA signal was also detected in the nuclei, suggesting potential, albeit less common, transcriptional regulatory effects that are likely minor in the context of this approach. These results validate the capability of PEI/PPI-based nanoparticles to target this pivotal brain region for therapeutic purposes, e.g., in PD.

Intriguingly, we found that the efficacy of PEI/PPI-based nanoparticles to knock down SNCA mRNA and protein *in vivo* does not necessarily depend on the level of nanoparticles in the brain, but rather on the polymer used. PEI has been previously used for intranasal application, including delivery of (PEI-conjugated) peptide drugs in an Alzheimer’s disease mouse model,^49^ the coating of inorganic nanoparticles for intranasal delivery,^50^ or for intranasal vaccination.^51^ To our knowledge, only one study has explored the use of PPI for intranasal delivery, and it demonstrated that PPI maltose dendrimers can successfully cross the blood-brain barrier and influence Aβ aggregation in a mouse model of Alzheimer’s disease.^52^ Currently, only two studies have investigated intranasal delivery of siRNAs or microRNAs (miRNAs) in the context of PD. One study explored the intranasal administration of small extracellular vesicles in mice with manganese-induced Parkinson-like symptoms, revealing that vesicle-associated miRNAs play a neuroprotective role. However, this study only examined miRNA overexpression *in vitro*.^53^ A second study used intranasal delivery of naked siRNA or antisense oligonucleotide conjugated with the triple monoamine reuptake inhibitor indatraline in transgenic mice expressing human wild-type aSyn. The results showed a 20%–40% reduction in aSyn expression in the brainstem monoamine nuclei.^54^ Notably, in the present study, the delivery of a low siSNCA dose using PEI/PPI-based nanoparticles, i.e., PPI-Y PP and LP10Y LPP, achieved a 70%–80% reduction in *SNCA* mRNA and a 50%–70% reduction in aSyn protein levels.

As a lipopolyplex, LP10Y even showed increasing knockdown efficacy over time *in vitro*, indicating the benefits of liposome addition (increased cellular uptake, less tendency to aggregate than polyplexes).^19^^,^^25^ Consistent with this, it was also very effective *in vivo* and reached the brain in larger quantities compared with the non-liposomal LP10Y. This confirms the benefits of the liposome additive previously observed in the case of PEI F25 LPP chosen for direct intracranial siRNA delivery in a syngeneic orthotopic mouse glioma model.^23^ Still, the chemical composition of the polymer influences biological LPP activities, as seen from the higher *in vitro* knockdown efficiency of the linear LP10Y LPP compared with the branched P10Y LPP that was also visible *in vivo*. Although there is high variability in mRNA *SNCA* levels in some treatment groups, our results were confirmed by measurements of alpha-synuclein protein levels, which showed a significant positive correlation with *SNCA* mRNA levels (*p* < 0.0001; r = 0.394). The LP10Y LPP efficiently reduced both SNCA mRNA and protein expression.

Notably, this effect was more robust in the case of the lower dose as compared with higher dose treatment. Thus, overall we observed that maximum achievable knockdown efficacies also proved to be dose-dependent, but in favor of the lower siSNCA dose. This is presumably due to some aggregation in case of the higher dose, as more nanoparticles occur within the same amount of brain tissue. The previously observed tendency of nanoparticles, particularly polyplexes, to form larger aggregates upon prolonged storage at room temperature or 37°C suggests that large, inactive particles are likely to be formed.^28^ Furthermore, the higher dose was administered at a double intranasally applied volume. This could be another reason for the reduced knockdown efficacy, as with increasing volume, more substance reaches the lung, whereas less remains in the head.^55^ Thy1-aSyn mice exhibit a brain region-dependent 3- to 10-fold overexpression of human *SNCA*.^16^ Consequently, the effective dose of siSNCA used in this study can be expected to be more than adequate to silence *SNCA* in human tissue from patients with sporadic PD expressing endogenous levels of *SNCA*, and thus indicates a sufficiently broad therapeutic window. Importantly, the nanocomplexes did not induce neuroinflammation characterized by increased reactive microglia or astrogliosis in the forebrain (olfactory bulb) and deeper brain regions (substantia nigra) of mice. The absence of any significant cytokine response in brain lysates further supports this statement. The very good *in vivo* biocompatibility of PPI-Y and LP10Y confirms previous findings that had already shown the absence of hepatotoxicity or nephrotoxicity after systemic application (intraperitoneal or intravenous) as well as the absence of any immune reactions.^24^ Together with their low cytotoxic potential *in vitro*, PEI/PPI-based nanoparticles thus demonstrate high biocompatibility in both *in vitro* and *in vivo* settings. Moreover, this PEI/PPI-based delivery approach is particularly attractive as it relies on chemically simple, cost-effective carriers and already approved liposomes (DPPC) with defined structures that are available on a large scale, as well as a non-invasive administration technique already practiced in human patients, which is of particular importance when it comes to broad medical applications.

Taken together, the tyrosine-modified LP10Y lipopolyplex and the PPI-Y dendriplex show particularly promising profiles as delivery platforms for RNAi (e.g., siRNA, miRNA), characterized by high gene knockdown efficacy, deep tissue penetration, and high biocompatibility in both SH-SY5Y neuroblastoma cells overexpressing wild-type aSyn^56^ and the Thy1-aSyn mouse model of PD. Our findings indicate that PEI/PPI-based nanoparticles represent efficient nose-to-brain delivery systems for RNAi therapy and offer a promising approach to suppress gene expression in the brain for the treatment of PD.

## Materials and methods

### Complexation of siRNA in PEIs/PPIs

SiRNA against human *SNCA* (siSNCA; Dharmacon Horizon Discovery, Cambridge, UK; sense, 5′-GCAGGAAAGACAAAAGAGGUU-3′, antisense, 5′-CCUCUUUUGUCUUUCCUGCUU-3′) or siRNA against luciferase serving as negative control (siCtrl; Dharmacon Horizon Discovery, Cambridge, UK; sense, 5′- CUUACGCUGAGUACUUCGAUU-3′, antisense, 5′- UCGAAGUACUCAGCGUAAGUU-3′) was used. Polymer synthesis and complexation of siRNAs was performed as described previously (LP10Y,^57^ P10Y,^20^^,^^58^ PEI F25,^25^^,^^34^ PPI-Y^24^^,^^26^). 1,2-dipalmitoyl-sn-glycero-3-phosphocholine (DPPC) (Avanti Polar Lipids, Alabaster, AL) liposomes were prepared by the thin film hydration and extrusion method as described in Ewe et al.^28^ In short, siRNA and P10Y, LP10Y, or PPI-Y polymers were each dissolved in HN buffer (150 mM NaCl, 10 mM HEPES; pH 7.4) for *in vitro* experiments and in trehalose buffer (10% trehalose, 20 mM 4-(2-hydroxyethyl)-1-piperazineethanesulfonic acid [HEPES]; pH 7.4) for *in vivo* experiments. For complexation of PEI F25, HBG buffer (5% glucose, 20 mM HEPES; pH 7.4) was used instead of trehalose buffer. SiRNA was dissolved in the same buffer as the corresponding polymer, added to the polymer solution, mixed by vortexing, and incubated for 30 min at room temperature (RT) for complexation of polyplexes. For complexation of lipopolyplexes, DPPC liposomes were added to the siRNA-polymer solution and incubated at RT for 5 min. Subsequently, lipopolyplexes were incubated at 50°C for 10 min in an ultrasonic bath and afterward incubated at RT for 60 min. Complex formation is based on electrostatic interactions between the positively charged polymers and the negatively charged siRNAs.^59^ Polymer/siRNA mass ratio was 2.5 and DPPC/polymer mass ratio was 10 for P10Y, LP10Y, and PPI-Y. PEI F25 was complexed with a polymer/siRNA mass ratio of 7.5 and a DPPC/polymer mass ratio of 5. In the context of *in vitro* uptake experiments, siCtrl labeled with the fluorescent dye Alexa Fluor 647 (AF647) was used. In terms of *in vitro* gene knockdown and cytotoxicity analyses, siSNCA and siCtrl were applied. For examining the distribution and cellular uptake of nanoparticles in the CNS *in vivo*, AF647-siCtrl (300 pmol) and unlabeled siSNCA (300 pmol), which functioned as a control, were utilized. During *in vivo* gene knockdown experimentation, siSNCA and siCtrl were used in doses of 300 pmol and 600 pmol. Naked siRNA was tested in preliminary experiments (see Figure S6); however, no knockdown of *SNCA* expression was detected in SH-SY5Y cells. Given its limited therapeutic relevance^11^^,^^60^ and the lack of effective preliminary results, naked siRNA was not included as a control in the *in vivo* experiments to reduce animal usage in alignment with the 3Rs principle.

### Nanoparticle characterization

The various (lipo-)polyplexes were characterized for siRNA complexation efficacies via agarose gel electrophoresis, particle sizes, and zeta potentials. To this end, siRNA complexes used for *in vitro* transfections were prepared in HN buffer as described above, at a final siRNA concentration of 1 μg/60 μL and a polymer/siRNA mass ratio 2.5 for all tyrosine-modified polymers and mass ratio 7.5 for PEI F25, respectively. Complexes used for *in vivo* applications were prepared in trehalose buffer (tyrosine-modified polymers) or HBG buffer (PEI F25) at a final siRNA concentration of 1 μg/10 μL using the same polymer/siRNA mass ratios as above.

For agarose gel electrophoresis, complexation mixes containing 0.2 μg siRNA were mixed with 10x siRNA loading dye (50% [v/v] glycerol, 0.1% [w/v] xylene cyanol in dH_2_O) and separated on a 1.5% agarose gel with ethidium bromide and TAE running buffer at 80 V for 15 min. Unbound siRNA appeared as bands that were visualized using the ODYSSEY Fc imaging system (LI-COR, Lincoln, NE, USA).

The hydrodynamic diameters and zeta potentials of the complexes were measured by dynamic light scattering (DLS) and electrophoretic light scattering (ELS), respectively, using the Zetasizer Advanced Series Ultra Red (Malvern Panalytical, Kassel, Germany). From complexes prepared as described above, a volume containing 5 μg siRNA was further diluted in 1 mL Millipore water in a 1-cm disposable cuvette. The sample measurements were performed at 25°C using the refractive index and viscosity of water. For the size measurements, the MADLS option (multi angle light scattering) at 173°, 90°, and 13° was used. For determining the zeta potential, the sample was filled into a capillary cell and measured using the monomodal analysis model. Data from at least three independent measurements were collected and processed using the ZS Xplorer software (v4.0.0).

### Uptake analysis in SH-SY5Y neuroblastoma cells

SH-SY5Y neuroblastoma cells (a kind gift from Imke Steffen, Research Center for Emerging Infections and Zoonoses, Hannover, Germany) were maintained in Iscove's Modified Dulbecco's Medium (IMDM; Thermo Fisher Scientific, Waltham, MA) supplemented with 10% fetal calf serum (FCS; Linaris, Dossenheim, Germany) and 1% penicillin/streptomycin (P/S; Merck, Darmstadt, Germany). Differentiation was performed for 5 days using 10 μM retinoic acid (Sigma-Aldrich, St. Louis, MO).

For uptake experiments, differentiated SH-SY5Y cells were seeded 24 h before transfection in eight-well chamber slides (density: 6.25 × 10^4^/cm^2^) with 500 μL culture medium IMDM. After incubation with AF647-labeled nanoparticles (50 pmol siRNA) for 4 h or 24 h at 37°C, cells were fixed with 4% paraformaldehyde, stained with Phalloidin iFluor 488 (Abcam, Milton, UK), coverslipped with mounting medium containing 4′,6-diamidino-2-phenylindole (DAPI; Thermo Fisher Scientific, Waltham, MA), and examined by fluorescence microscopy.

### *SNCA* knockdown analysis in SH-SY5Y neuroblastoma cells

SH-SY5Y tetracycline (Tet)-Off aSyn-overexpressing cells (a kind gift from Tiago Fleming Outeiro, Department of Experimental Neurodegeneration, Göttingen, Germany and Leonidas Stefanis, Biomedical Research Foundation of the Academy of Athens, Athens, Greece) were maintained in Roswell Park Memorial Institute 1640 Medium (RPMI; Merck, Darmstadt, Germany) supplemented with 10% FCS and 1% P/S. Selection and maintenance of clones inducibly overexpressing aSyn was performed using hygromycin B (50 μg/mL; Sigma-Aldrich, St. Louis, MO) and geneticin (G418, 500 μg/mL; Thermo Fisher Scientific, Waltham, MA). Alpha-synuclein overexpression is switched off in the presence of doxycycline hydrochloride (1 μg/mL; Merck, Darmstadt, Germany) and induced by removal of doxycycline from the medium. Overexpression lasted for 10 days in culture before cells were seeded for experiments. Stock cultures were kept in the presence of doxycycline (see effects of doxycycline in Figure S6). For determination of *SNCA* mRNA levels, cells were seeded 24 h before transfection experiments in six-well plates (density: 2.6 × 10^4^/cm^2^) cultured with 2 mL RPMI, and transfected with 75 pmol siRNA/well in biological triplicates. Cells were harvested at specified time points (24–120 h). *SNCA* mRNA levels were determined by quantitative reverse transcription polymerase chain reaction (qRT-PCR) as described in the following sections.

### Cytotoxicity analysis by lactate dehydrogenase release assay

SH-SY5Y Tet-off aSyn-overexpressing cells used for lactate dehydrogenase (LDH) assay were seeded in 96-well plates (density: 1.56 × 10^5^ cm^2^) in triplicates with 100 μL RPMI, transfected with nanoparticles (15 pmol siRNA/well) and incubated for 24–72 h. Cytotoxicity was examined by measuring LDH release into the supernatant of transfected cells (LDH assay; Thermo Fisher Scientific, Waltham, MA). Percent proportion of LDH release was normalized to cells treated with 1x Triton for 1 h (100% cell death).

### Animals

Mice overexpressing human wild-type *SNCA* under the murine Thy1-promoter (Thy1-aSyn mice, Masliah-line 61)^16^^,^^17^ maintained on a hybrid C57BL6/DBA2 background were used. The genotypes of all Thy1-aSyn mice were determined at 2 weeks of age and confirmed at the end of the experiment by PCR amplification analysis of ear DNA. Two-month- or six-month-old male transgenic mice from multiple litters were used in this study. Female Thy1-aSyn transgenics are not suitable for this study due to the insertion of the transgene into the X chromosome, from which one copy is randomly and irreversibly inactivated leading to low and variable transgene expression. Mice used in this study were in-house bred and grouped of up to six individuals in Makrolon type III cages (BIOSCAPE/Ebeco, Castrop-Rauxel, Germany) equipped with standard bedding (shredded wood), nesting material, wooden chew sticks, and red plastic houses for environmental enrichment. Animals were housed under controlled environmental conditions (room temperature 22 ± 2°C, relative humidity 50%–60%; values were recorded during the daily animal check) with a reverse 12-h light/dark cycle (lights off at 11:00 a.m.). Standard laboratory chow (Altromin 1324 standard diet) and tap water were available *ad libitum*.

For intranasal application, animals were held with a skin grip for a short time. Using a conventional micropipette, small droplets were placed onto the nostrils to be inhaled by the animal. The dose selection was based on previous experiments in which amounts of 10 μg siRNA complexed in PEI-based nanoparticles per systemic (intraperitoneal) administration have been found to be efficient for profound gene knockdown in mice.^19^^,^^20^^,^^57^ In addition, pilot tests using siRNA complexed with PEI-based lipopolyplexes and administered intranasally (5 days, once daily, 2 μg of siRNA in 20 μL/nostril) revealed a distribution of the nanoparticles across the CNS in Thy1-aSyn mice. In the present study, mice received daily applications of nanoparticles in a dosage of either 300 pmol siRNA/day (4 μg in 20 μL/nostril) or 600 pmol siRNA/day (8 μg in 40 μL/nostril) on 4 consecutive days and were euthanized 24 h after the last application. Mice were euthanized by an intraperitoneal overdose of pentobarbital (800 mg/kg; Euthadorm, CP-Pharma-Handelsgesellschaft GmbH, Burgdorf, Germany) in a volume of 1 mL/kg followed by transcardial perfusion.

### Ethics statement

All animal experiments were conducted in compliance with the European Union council directive 2010/63/EU and the German Animal Welfare Act. They were approved by the by the animal subjects review board of the Lower Saxony State Office for Consumer Protection and Food Safety (LAVES, Oldenburg, Germany; file numbers: 20/3376 and 20/3526). All animal experiments of this study were conducted and are reported in accordance with ARRIVE guidelines. Experiments were designed to minimize animal numbers and suffering.

### Immunohistochemical analysis of CNS distribution and cellular uptake in Thy1-aSyn mice

For analysis of CNS distribution of nanoparticles, mice were perfused with phosphate-buffered saline (PBS) followed by 4% formaldehyde. Whole brains were removed immediately, fixed for 48 h in 4% formaldehyde, cryoprotected in a 10%–30% gradient of sucrose in 0.1 M PBS and stored at 4°C. Brains were cut in 40-μm serial coronal sections using a cryostat and stored free-floating at −20°C. Sections were blocked in goat blocking solution for 1 h, incubated with monoclonal rabbit anti-Lamp1 primary antibody (Abcam, Milton, UK), monoclonal mouse anti-TH primary antibody (Immunostar, Hudson, WI), monoclonal guinea pig anti-NeuN primary antibody (Synaptic Systems, Göttingen, Germany) diluted 1:500, and monoclonal rabbit anti-beta-III-tubulin primary antibody (Abcam, Milton, UK) diluted 1:1,500 in tris-buffered saline (TBS)/2% normal goat serum/BSA/0.5% Triton X- at 4°C overnight. Negative control sections were incubated in the same solution without primary antibody. Sections were washed in TBS and then incubated with secondary antibodies AF405-labeled anti-rabbit IgG, AF488-labeled anti-mouse IgG, AF488-labeled anti-rabbit IgG, and AF555-labeled anti-guinea pig IgG (Thermo Fisher Scientific, Waltham, MA) at 1:500 dilution in TBS/2% normal goat serum/BSA/0.5% Triton X- at RT for 1 h protected from light. Sections were washed in TBS, treated with TrueBlack Lipofuscin Autofluorescence Quencher (Cell Signaling, Danvers, MA) according to the manufacturer’s protocol and coverslipped with mounting medium (Thermo Fisher Scientific, Waltham, MA).

For quantification of ionized calcium-binding adapter molecule 1 (Iba1) and glial fibrillary acidic protein (GFAP) signal, and for *SNCA* mRNA and aSyn as well, mice were perfused with 0.1 M PBS, brains were removed immediately and separated into the two hemispheres. One hemisphere was flash frozen in liquid nitrogen and stored at −80°C for molecular analyses. The anterior half of the hemisphere was used for western blot analysis, the posterior half for qRT-PCR. The other hemisphere was used for immunohistochemical analyses and was therefore postfixed in 4% formaldehyde for 48 h, cryoprotected in a 10%–30% gradient of sucrose in 0.1 M PBS and stored at 4°C. Brains were cut in 40-μm serial coronal sections using a cryostat and stored free-floating at −20°C. For quantification of Iba1 and GFAP, sections were processed for immunohistochemistry. Staining was performed as described above by using the following antibodies: monoclonal rat anti-GFAP primary antibody (Thermo Fisher Scientific, Waltham, MA) and monoclonal rabbit anti-Iba1 primary antibody (Abcam, Milton, UK); secondary antibodies AF488-labeled anti-rat IgG (Thermo Fisher Scientific, Waltham, MA) and AF647-labeled anti-rabbit IgG (Thermo Fisher Scientific, Waltham, MA). Sections were washed in TBS and coverslipped with mounting medium containing DAPI (Thermo Fisher Scientific, Waltham, MA).

A Zeiss Axio Observer 7 fluorescence microscope using either 20× or 40× magnification lenses and a Zeiss Colibri 7 LED light source coupled with a Zeiss Zen Pro software (Carl Zeiss, Oberkochen, Germany) was used. For confocal imaging a Zeiss LSM 980 with Airyscan2 (Carl Zeiss, Oberkochen, Germany) at the Research Core Unit for Laser Microscopy at the Medical School Hannover, Germany, with a 25× magnification lens was used for visualization and image acquisition. Image analysis was performed using ImageJ (National Institutes of Health, Bethesda, MD) on representative images for each region of interest. Five olfactory bulb slices, three prefrontal cortex slices, and three substantia nigra slices per animal were used to quantify nanoparticle distribution.

### Quantification of *SNCA*, *IL-1β*, *IL-6,* and *TNF-α* mRNA expression by qRT-PCR

RNA was extracted according to the manufacturer’s protocol using the RNeasy Plus Mini Kit (QIAGEN, Venlo, Netherlands). Concentration and purity of RNA was determined using a NanoDrop One spectrophotometer (Thermo Fisher Scientific, Waltham, MA). First-strand complementary (c)DNA synthesis was performed using the iScript Reverse Transcription Supermix (Bio-Rad, Hercules, CA) and a T100 Thermal Cycler (Bio-Rad, Hercules, CA).

Specific TaqMan probes (Thermo Fisher Scientific, Waltham, MA) were mixed with SsoAdvanced Universal Probes Supermix (Bio-Rad, Hercules, CA) to obtain a reaction mix. Two microliters containing 200 ng of cDNA were added to the reaction mix for a total reaction volume of 10 μL per well, triplicates were used and amplification was performed on a CFX Connect Real-Time PCR detection system (Bio-Rad, Hercules, CA). GeNorm (https://genorm.cmgg.be/) was used to analyze candidate reference genes *actb*, *gapdh*, and *hprt* according to average expression stability. *Actb* was analyzed as the most stable housekeeping gene and therefore used for normalization. For *SNCA* quantification, further normalization was done by setting the mean of siCtrl-treated animals to 100%.

### Quantification of aSyn expression by western blot analysis

Brain tissue and cells were lysed in radioimmunoprecipitation assay (RIPA) buffer (20 mM Tris, 50 mM NaCl, 0.5% [w/v] sodium deoxycholate, 0.5% [v/v] Triton X-100, pH 8.0) supplemented with complete protease inhibitor and phosSTOP (Roche, Mannheim, Germany) on ice by using a pestle and a syringe. Lysates were centrifuged and protein concentration of the supernatant was determined using the Pierce BCA Protein Assay Kit (Thermo Fisher Scientific, Darmstadt, Germany). Western blotting was performed by sodium dodecyl sulfate-polyacrylamide gel electrophoresis. Proteins were fractionated on 4%–20% Mini-PROTEAN TGX Stain-Free gels (Bio-Rad, Hercules, CA) and transferred to a polyvinylidene fluoride (PVDF) membrane. The membrane was blocked in EveryBlot Blocking Buffer (Bio-Rad, Hercules, CA) for 5 min and incubated with mouse anti-Syn1 monoclonal primary antibody (BD Biosciences, Heidelberg, Germany) or mouse anti-Syn211 monoclonal primary antibody (Abcam, Milton, UK) at a 1:500 dilution in blocking buffer at RT for 1 h. Then, the membranes were washed with PBST (PBS+0.05% Tween 20) and incubated with goat anti-mouse polyclonal secondary antibody (1:1,000; Agilent Dako, Santa Clara, CA) at RT for 1 h.

After washing, protein bands were visualized using SuperSignal West Femto Chemiluminescent Substrate (Thermo Fisher Scientific, Darmstadt, Germany) and the Chemidoc MP Imaging System (Bio-Rad Laboratories, Munich, Germany). Protein bands were quantified using ImageLab version 6.1.0 (Bio-Rad, Hercules, CA). Protein expression is presented as intensity of target protein bands normalized to total protein visualized by stain-free technology (Bio-Rad, Hercules, CA) (see Figure 4). Further normalization was done by setting the mean of siCtrl-treated animals to 100%.

### Statistical analysis

GraphPad Prism 10 (GraphPad, La Jolla, CA) was used for statistical evaluations. Normally distributed data were compared using two-way repeated measures analysis of variance (ANOVA) with the Geisser-Greenhouse correction followed by a Sidak correction for multiple comparisons (*in vitro* gene knockdown and cytotoxicity analyses) or unpaired t test with Welch’s correction in case of unequal variances (for *in vivo* gene knockdown experiments). All data were tested two-sided and the level of significance was set to α = 0.05.

## Data availability

The data that support the findings of this study are available from the corresponding author upon reasonable request.

## Acknowledgments

We thank Edith Kaczmarek, Sonja Stelz, Ivo Wiesweg, Pia Hollasch, Antonia Joseph, Andrea Ofner, Martina Gramer, Mandy Behnke, Maike Krüger, and Natalie Beiderwellen for excellent technical assistance at the University of Veterinary Medicine Hannover. Open Access funding was enabled and organized by Projekt DEAL. This project was funded in part by the 10.13039/501100005972Deutsche Krebshilfe (A.A., project 70115169).

## Author contributions

Funding acquisition, F.R. and A.A. Conceptualization, F.R., A.A., and M.F. Resources, F.R., A.A., A.E., T.F.O., and L.S. Methodology, A.A., A.E., F.R., T.F.O., L.S., and B.G. Investigation, Formal Analysis, and Visualization, I.D., S.W., and M.F. Writing – original draft, M.F., I.D., and S.W. Writing – review & editing, A.A., F.R., A.E., M.F., I.D., S.W., T.F.O., L.S., and B.G. Project administration, M.F., I.D., and F.R. Supervision, F.R., A.A., M.F., and A.E. F.R. directly accessed and verified the underlying data reported in the manuscript. All authors read and approved the final version of the manuscript.

## Declaration of interests

The authors declare no competing interests.

## Supplemental information

Document S1. Figures S1–S7

Document S2. Article plus supplemental information
