# Supplementary material for: Nose-to-brain siRNA delivery by PEI/PPI-based nanoparticles reduces α-synuclein expression in a Parkinson’s disease mouse model
Source: Mol Ther Nucleic Acids. 2025 Aug 6;36(3):102671. doi: 10.1016/j.omtn.2025.102671 (PMC12447568; doi:10.1016/j.omtn.2025.102671)
Supplement: Document S1. Figures S1–S7 [file mmc1.pdf]

OMTN, Volume *36*

Supplemental information

Nose-to-brain siRNA delivery by PEI/PPI-based nanoparticles reduces α-synuclein expression in a Parkinson's disease mouse model

Malte Feja, Isabell Drath, Sandra Weiß, Alexander Ewe, Birthe Gericke, Tiago F. Outeiro, Leonidas Stefanis, Achim Aigner, and Franziska Richter

Supplemental information

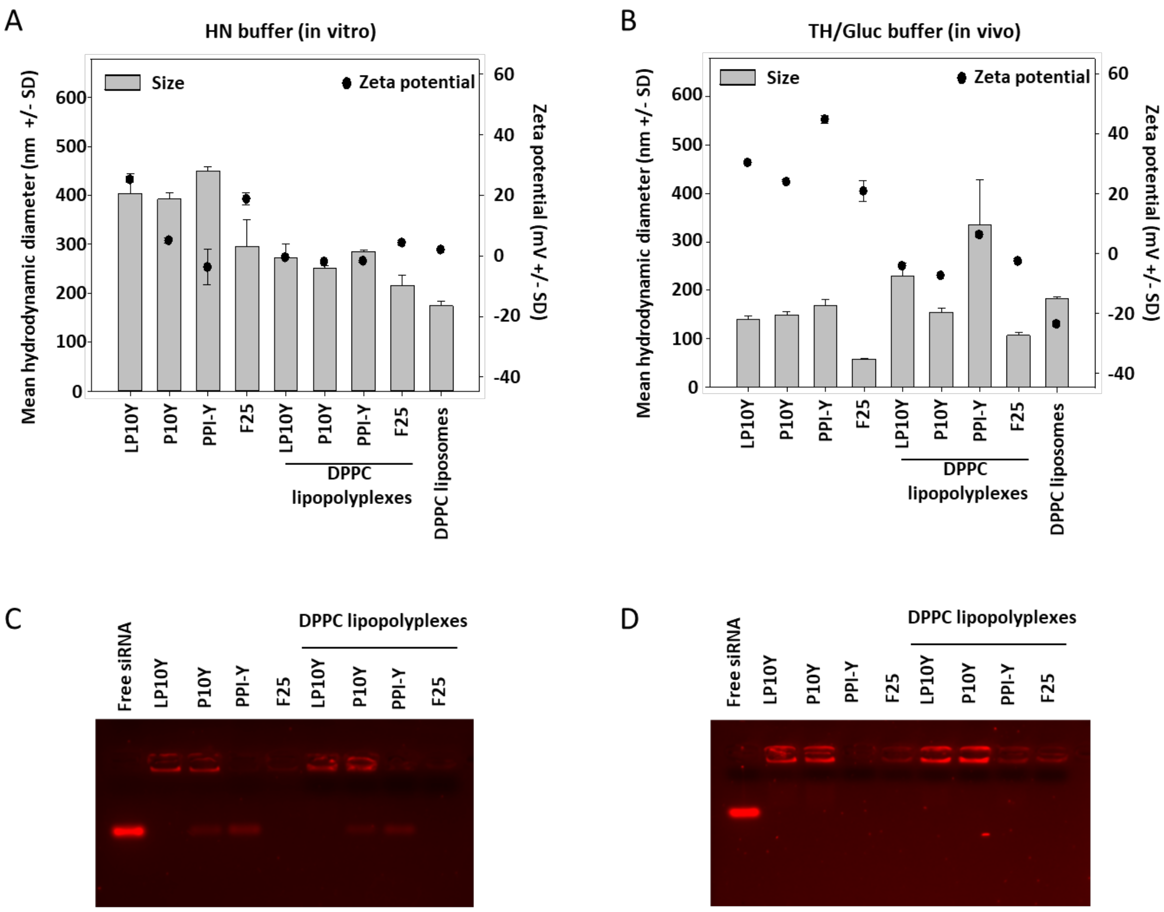

Figure S1: Physicochemical characterization of polyethylenimine (PEI)/polypropylenimine (PPI)-based nanoparticles. Hydrodynamic diameters (bars), as determined by dynamic light scattering (DLS), and zeta potentials (dots) measured by phase analysis light scattering (PALS) are shown of complexes prepared with the different polymers in the presence of HN buffer (A) or trehalose/glucose buffer (B) for complexation. At the polymer/siRNA ratios selected for complex formation, full complexation is achieved as demonstrated in agarose gel electrophoresis by the absence of the free siRNA band in the nanoparticle samples in the presence of HN buffer (C) or trehalose/glucose buffer (D).

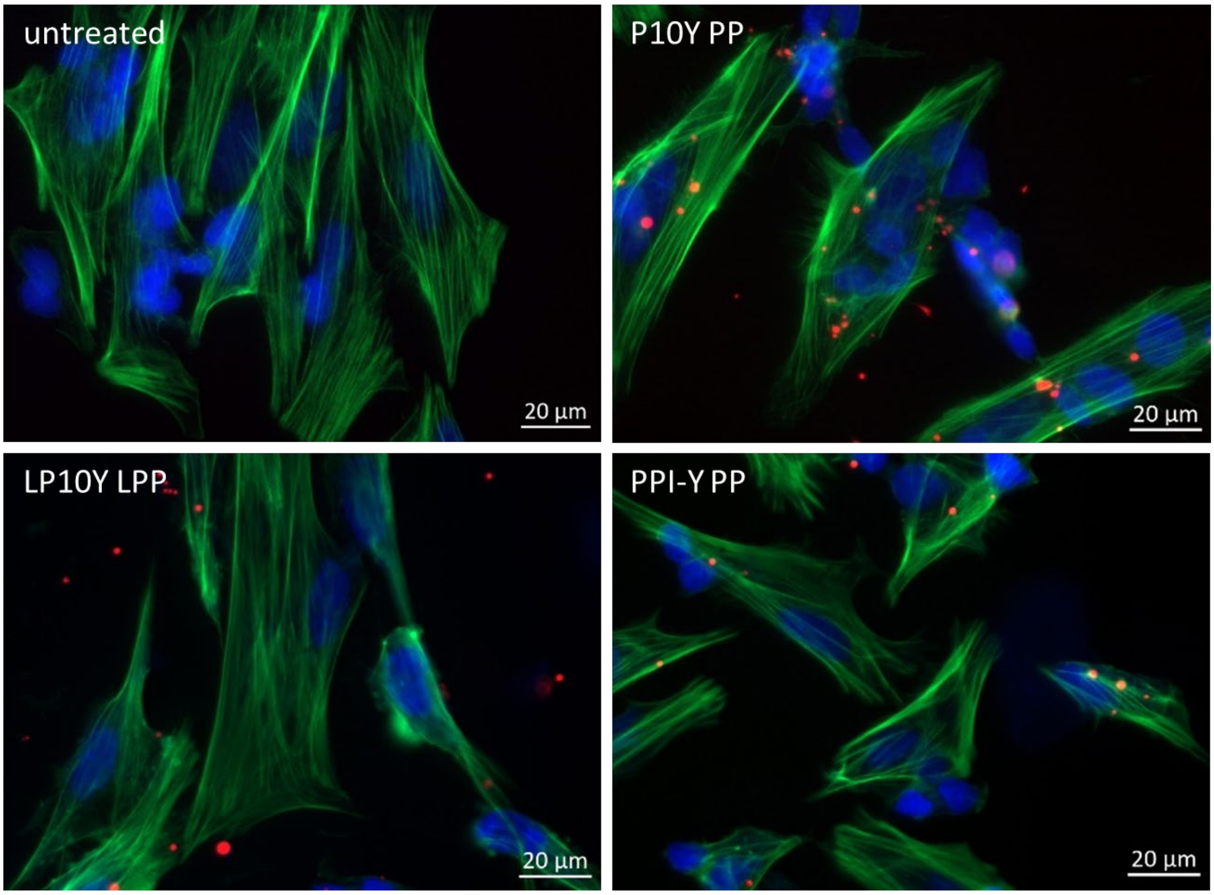

Figure S2: Cellular uptake of polyethylenimine (PEI)/polypropylenimine (PPI)-based nanoparticles *in vitro*. Representative immunofluorescence photomicrographs of differentiated SH-SY5Y neuroblastoma cells after 4-h incubation with AlexaFluor647-labeled small interfering RNA (AF647-siRNA; 50 pmol) complexed with tyrosine-modified branched (P10Y) and linear PEIs (LP10Y) or with tyrosine-modified PPI (PPI-Y) into polyplexes (PP) or lipopolyplexes (LPP) or without nanoparticles (untreated control). Red, AF647-siRNA PP/LPP; green, phalloidin-stained cytoskeleton; blue, DAPI-stained DNA; 630x total magnification; scale bar: 20 µm.

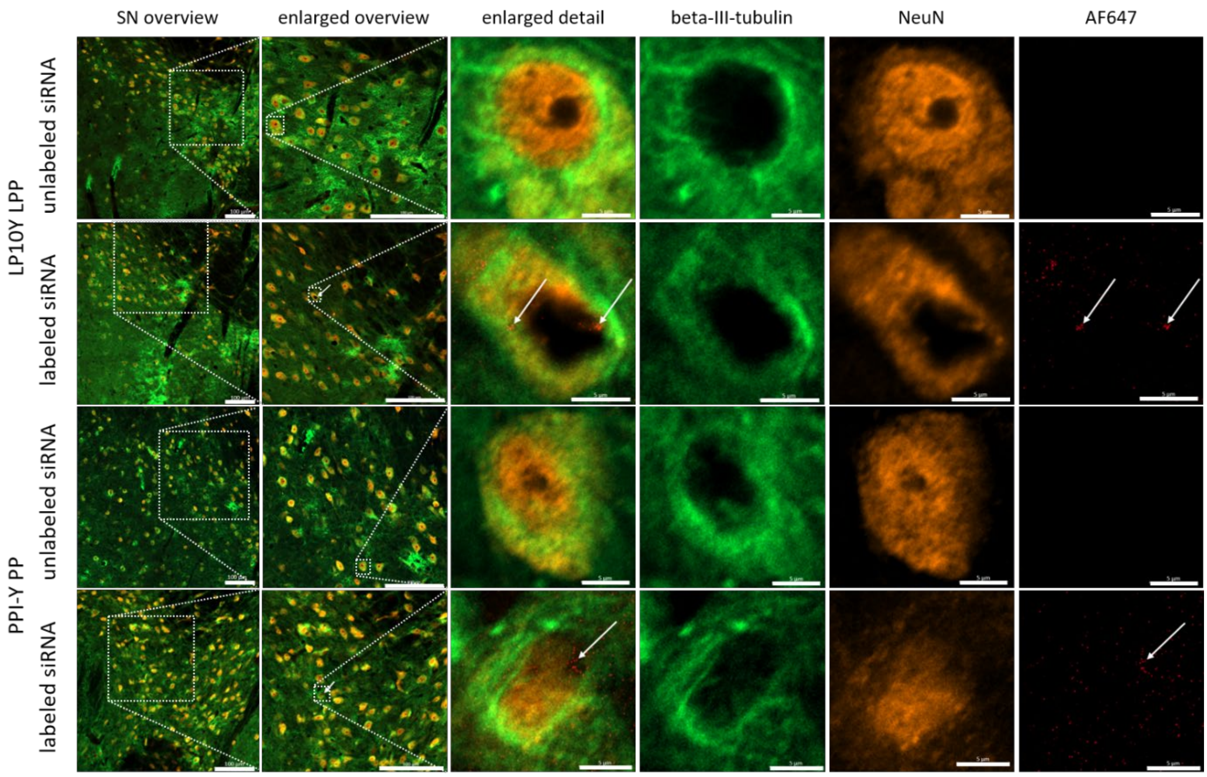

Figure S3: Neuronal uptake of polyethylenimine (PEI)/polypropylenimine (PPI)-based nanoparticles in the central nervous system (related to Figure 3). Alexa Fluor (AF) 647-labeled small interfering (si)RNA was complexed into tyrosine-modified linear PEI lipopolyplexes (LP10Y LPP) or tyrosine-modified PPI polyplexes (PPI-Y PP). Confocal microscopy of coronal brain sections visualized fluorescence of AF647-conjugated control small interfering RNA (siCtrl, 300 pmol) loaded into PPI-Y PP and LP10Y LPP in neurons of the substantia nigra (SN), indicating uptake into neuronal nuclei after intranasal administration (4 days, once daily) in mice. Negative control, nigral sections of mice treated with unlabeled siRNA-LP10Y/PPI-Y complexes showed no fluorescent signal in the far-red spectrum. Green, beta-III-tubulin as marker for neuronal axons, dendrites and soma; orange, NeuN as marker for neuronal nuclei; 250x total magnification; scale bar: 5 µm (overview image: 100 µm). White arrows indicate location of AF647-siRNA.

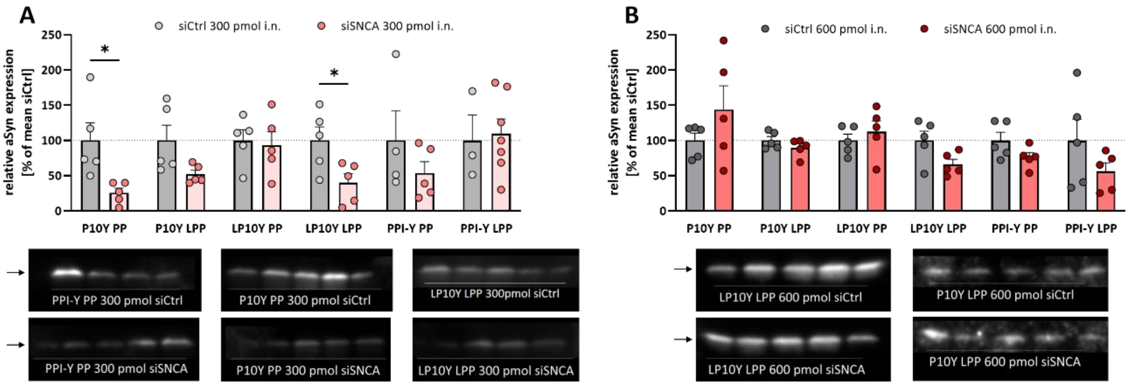

Figure S4: Knockdown of SNCA mRNA and alpha-synuclein (aSyn) by siRNA targeting the mRNA of aSyn (siSNCA) complexed into polyethylenimine (PEI)/polypropylenimine (PPI)-based nanoparticles (related to Figure 3). Control small interfering RNA (siCtrl) or siSNCA were loaded into tyrosine-modified branched (P10Y) and linear PEI (LP10Y) or tyrosine-modified PPI (PPI-Y) polyplexes (PP) or lipopolyplexes (LPP). The effects of intranasal application (once daily for 4 days) of nanoparticles loaded with 300 pmol (A) and 600 pmol siRNA (B) on aSyn expression in relation to total protein in the anterior half of the brain (see also Figures 3, S5) are shown as mean +SEM. Results are presented as relative values calculated as percent of mean siCtrl. Immunoblots with Syn211 as primary antibody and bands corresponding to aSyn (arrows at 17 kDa) are shown for respective treatment groups. Unpaired t-tests with Welch`s correction in case of unequal variances; $^{*}p < 0.05$.

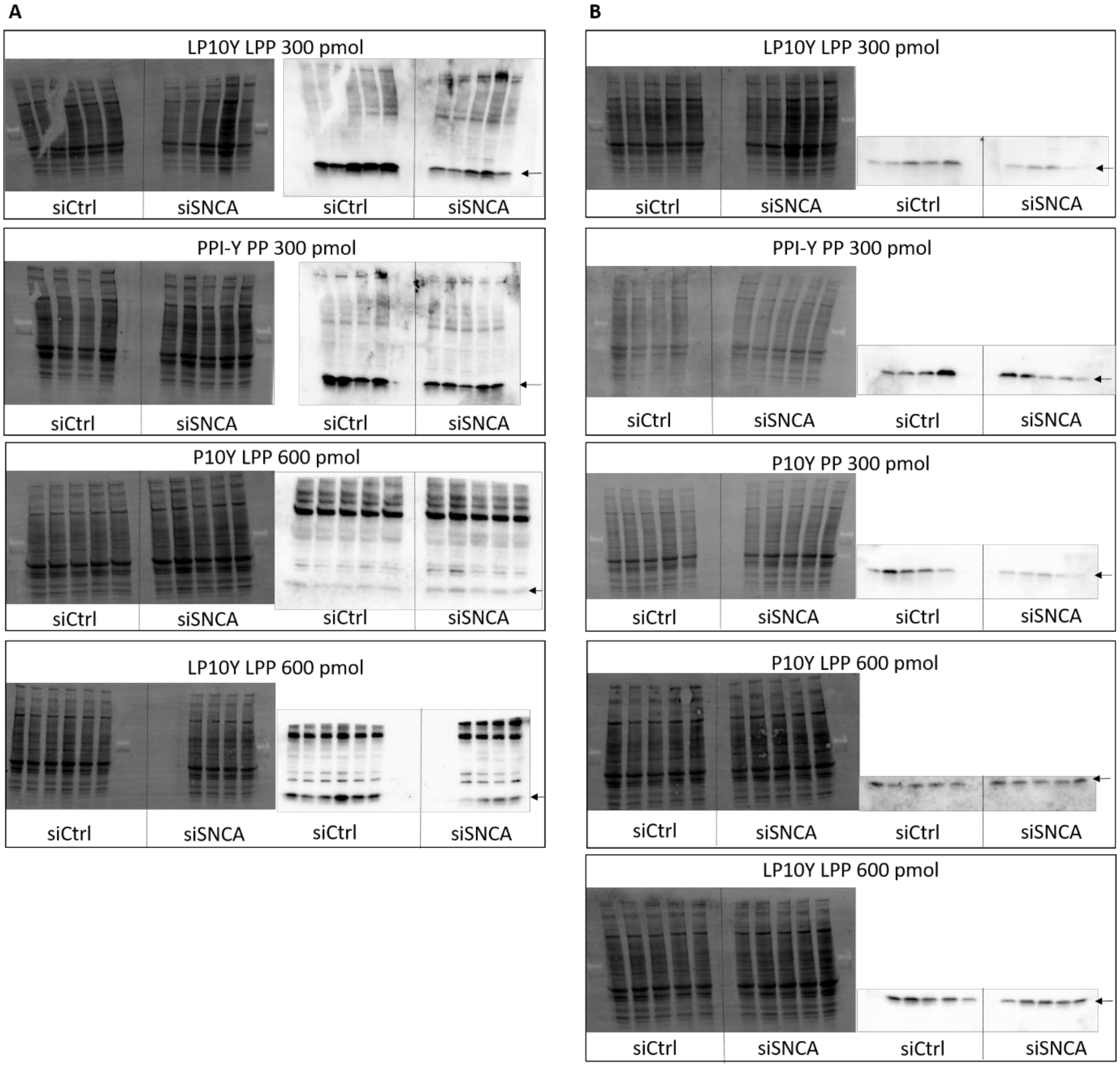

Figure S5: Alpha-synuclein (aSyn) Western blots from brain tissue samples (related to Figure 3 and S4) and corresponding total protein membranes. Immunoblots with Syn1 (A) or Syn211 (B) as primary antibody and bands corresponding to aSyn (arrows at 17 kDa) are shown in right columns. Membranes (left columns) for analysis of Syn211 (B) were cut right above 17 kDa before antibody incubation. Brain samples were treated with small interfering RNA (siRNA, 300 pmol and 600 pmol), either control siRNA (siCtrl) or siRNA targeting the mRNA of aSyn (siSNCA), loaded into tyrosine-modified branched (P10Y) and linear PEI (LP10Y) or tyrosine-modified PPI (PPI-Y) polyplexes (PP) or lipopolyplexes (LPP). Western blotting was performed by sodium dodecyl sulfate-polyacrylamide gel electrophoresis. Proteins were fractionated on 4–20% Mini-PROTEAN TGX Stain-Free gels and transferred to a polyvinylidene fluoride membrane.

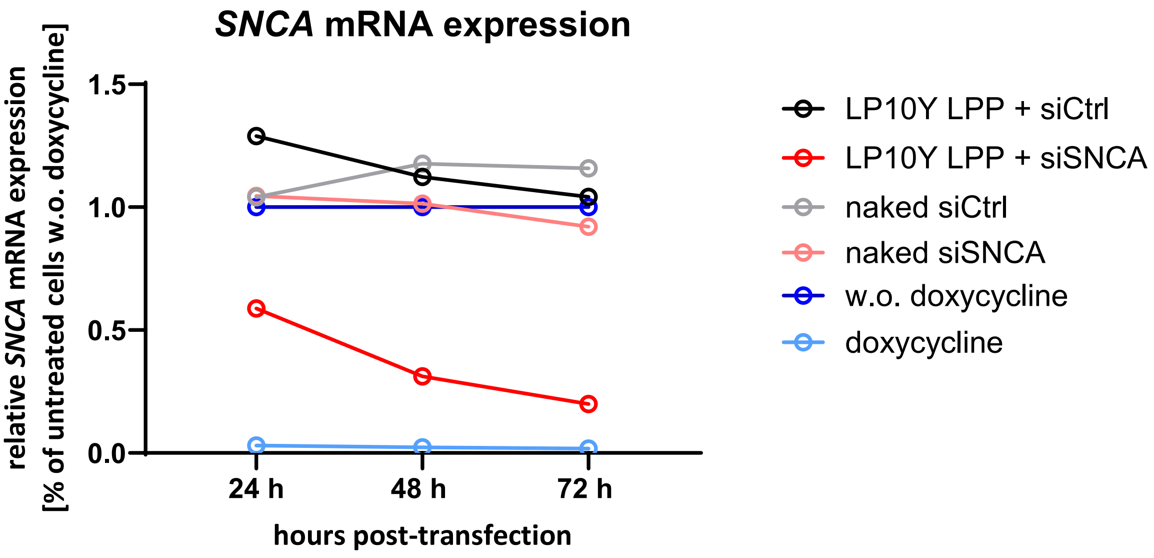

Figure S6: *SNCA* mRNA expression measured by RT-qPCR from SH-SY5Y tetracycline (Tet)-Off aSyn overexpressing cells. The effects of naked siRNA vs. siRNA-loaded nanoparticles on *SNCA* mRNA expression 24, 48, and 72 h after transfection are shown. Untreated cells with alpha-synuclein overexpression switched off by doxycycline are shown in light blue, alpha-synuclein overexpression switched on by removal of doxycycline from the medium is shown in dark blue. Treated cells were incubated with either naked control small interfering RNA (siCtrl, grey), naked siRNA targeting the mRNA of aSyn (siSNCA, salmon), siCtrl complexed with tyrosine-modified linear polyethyleneimine (PEIs, black) or siSNCA complexed with tyrosine-modified linear PEIs (red). Cells were seeded in 6-well plates with 250.000 cells per well and treated with 75 pmol siRNA per well. Results are presented as relative values calculated as percentage of mean untreated cells without doxycycline, using *actb* as reference gene.

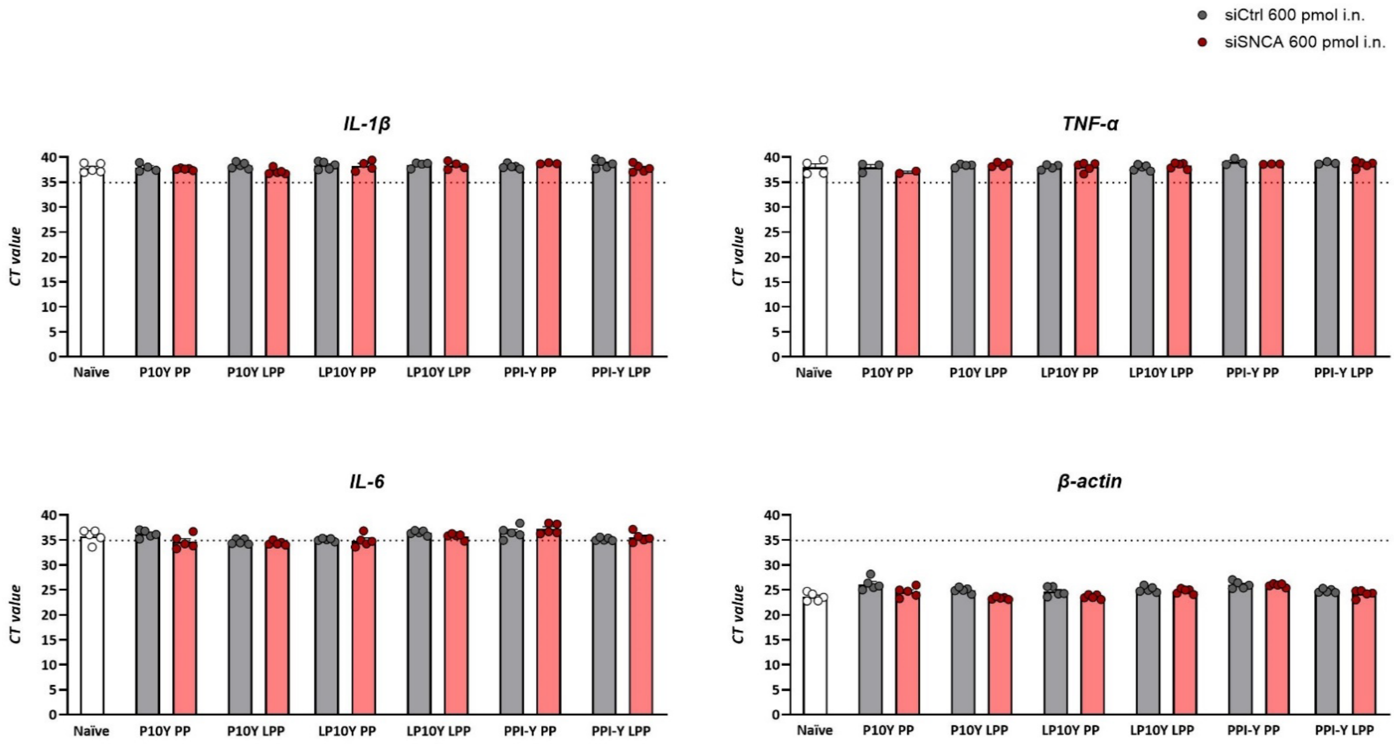

Figure S7: Expression of cytokine mRNA in brain tissue samples. Cycle threshold (CT) values of *IL-1β, IL-6, TNF-α* and housekeeping gene *β-actin* were measured by RT-qPCR in brain tissue of naïve mice and mice treated intranasal with siRNA targeting the mRNA of aSyn (siSNCA; red) or control small interfering RNA (siCtrl; grey) complexed into polyethylenimine (PEI)/polypropylenimine (PPI)-based nanoparticles. CT values shown are means +SEM of technical triplicates. Five samples were measured per group (see β-actin). For some cytokines, mRNA levels were below the detection limit in individual samples; therefore, the number of data points shown per group for cytokine measurements varies between 2 and 5. The dotted line indicates a CT > 35, which is considered as no template (cytokine mRNA) present.
